# Supplementary material for: Detection of Clinical and Subclinical Lumpy Skin Disease Using Ear Notch Testing and Skin Biopsies
Source: Microorganisms. 2021 Oct 19;9(10):2171. doi: 10.3390/microorganisms9102171 (PMC8541182; doi:10.3390/microorganisms9102171)
Supplement: Supplementary file 1 [file microorganisms-09-02171-s001.zip › microorganisms-1355067-supplementary.pdf]

**Table S1:** The immunoperoxidase monolayer assay (IPMA) scoring for all animals in both animal experiments. The IPMA scoring is expressed as strong positive, positive or weak positive and indicated with a colour code as shown at the bottom of the table.

| | R01 | | R02 | | R03 | | R04 | | R05 | | R06 | | R07 | | R08 | | R09 | | R10 | |
|---|---|---|---|---|---|---|---|---|---|---|---|---|---|---|---|---|---|---|---|---|
| | 1/50 | 1/300 | 1/50 | 1/300 | 1/50 | 1/300 | 1/50 | 1/300 | 1/50 | 1/300 | 1/50 | 1/300 | 1/50 | 1/300 | 1/50 | 1/300 | 1/50 | 1/300 | 1/50 | 1/300 |
| 0 dpi | N | N | N | N | N | N | N | N | N | N | N | N | N | N | N | N | N | N | N | N |
| 5 dpi | N | N | N | N | N | N | N | N | N | N | N | N | N | N | N | N | N | N | N | N |
| 6 dpi | N | N | N | N | N | N | N | N | N | N | N | N | N | N | N | N | N | N | N | N |
| 7 dpi | N | N | N | N | N | N | N | N | N | N | N | N | N | N | N | N | N | N | N | N |
| 8 dpi | N | N | P | N | N | N | N | N | N | N | N | N | N | N | N | N | N | N | N | N |
| 9 dpi | P | N | P | N | P | N | N | N | P | N | N | N | N | N | N | N | N | N | N | N |
| 12 dpi | P | P | P | P | P | P | P | N | P | P | P | P | P | P | P | N | P | P | P | P |
| 14 dpi | P | P | P | P | P | P | P | N | P | P | P | P | P | P | P | N | P | P | P | P |
| 16 dpi | P | P | † | † | P | P | P | N | P | P | P | P | P | P | P | P | P | P | P | P |
| 19 dpi | P | P | † | † | P | P | P | P | P | P | P | P | P | P | P | P | P | P | P | P |
| 21 dpi | P | P | † | † | P | P | P | P | P | P | P | P | † | † | † | † | † | † | P | P |

P Strong Positive   P Positive   P Weak positive   N Negative   † Euthanasia
